# Quantification of surgical trauma: comparison of conventional and minimally invasive surgical techniques for pertrochanteric fracture surgery based on markers of inflammation (interleukins)

**DOI:** 10.1007/s10195-012-0199-6

**Published:** 2012-06-13

**Authors:** F. del Prete, T. Nizegorodcew, P. Regazzoni

**Affiliations:** 1 Departement of Surgery, ASL10, Ospedale San Giovanni di Dio, Florence, Italy; 2 Department of Orthopaedic Sciences and Traumatology, University Hospital Agostino Gemelli, Catholic University, Rome, Italy; 3 University Hospital Basel, Spitalstrasse 21, Basel, Switzerland

**Keywords:** Quantification of surgical trauma, Measuring surgical trauma, Gliding hip screw, Minimally invasive technique, Interleukins

## Abstract

**Background:**

Fixation of pertrochanteric fracture is undoubtedly an additional trauma after the fracture itself. In elderly patients, it might have an important impact on the whole organism. In the literature we find various techniques to perform this type of surgery. Up to now, there are no parameters validated for quantification of the invasiveness of a surgical procedure; it is therefore still not demonstrated that any method is less invasive than any other. In an effort to find a way to quantify the invasiveness of a surgical procedure, inflammatory markers were collected in patients undergoing fixation of trochanteric fracture with gliding hip screw [dynamic hip screw (DHS)] using either a conventional (DHS conv) or minimally invasive (DHS MIO) technique.

**Method:**

Two groups of patients were investigated prospectively; 36 of them were treated with conventional DHS technique and 32 with minimally invasive technique. Mean age was 84.7 ± 7.20 and 82.78 ± 7.71 years, respectively. Fracture type was classified according to the AO classification. Interleukin (IL)-6, IL-10, IL-8, and tumor necrosis factor (TNF)-α were measured 1 h before and 1 h after surgery. Student’s *t* test, chi-square test, and multivariate logistic regression were used for statistical analysis.

**Results:**

Preoperative levels of interleukins showed no significant differences between the two groups. In contrast, the postoperative blood level of IL-6 in patients operated with DHS conv technique (78.41 ± 67.04 pg/ml) was on average higher than in patients operated by DHS MIO technique (39.02 ± 37.36 pg/ml), the mean difference being 39.39 pg/ml [95 % confidence interval (CI) 12.65–66.13 pg/ml; *p* = 0.0045]. Multivariate logistic regression (backward method with limit of significance 0.05) confirmed that patients operated by conventional technique were significantly more likely to have increased IL-6 after surgery than those operated by MIO technique. IL-8 was measured in only 36 patients (20 for DHS conv, 16 for DHS MIO). No significant differences were found between the two groups; however, there was a drastic decrease postoperatively (*p* < 0.0001) regardless of the type of surgery performed. IL-10 and TNF-α were tested in all subjects, but did not show significant differences between the two groups. Average length of incision was significantly different (4.61 cm, 95 % CI 3.50–5.71 cm; *p* < 0.001) between the two groups, being 11.65 ± 2.64 cm for DHS conv and 7.05 ± 1.77 cm for DHS MIO. Similarly, average units of red blood cells (RBCs) transfused [performed for hemoglobin (Hb) <9 g/dl and/or hematocrit (HCT) <27 %] was higher (2.22 ± 0.99) in the DHS conv group compared with the DHS MIO group (1.09 ± 1.20), with average difference of 1.13 (95 % CI 0.59–1.66; *p* < 0.001).

**Conclusions:**

This attempt to quantify the invasiveness of internal fixation for trochanteric fracture comparing two techniques (DHS conv versus DHS MIO) based on inflammatory markers (IL-6) has given encouraging results. Measurement of systemic inflammatory response to local tissue damage caused by osteosynthesis using IL-6 as marker seems to confirm the lower invasiveness of MIO techniques. These results for trauma cases seem in line with those published for hip prosthesis. Ongoing further studies analyzing the effect of nailing will confirm or invalidate these preliminary results.

## Introduction

Pertrochanteric fracture represents one of the most common injuries with absolute indication for surgery. The goals of treatment are fracture reduction, which can be performed with either open or closed techniques, and stabilization allowing early patient mobilization. For these patients, mainly elderly with multiple comorbidities, surgery represents the “second hit” after the first fracture trauma. Minimally invasive methods for indirect reduction and fixation [1, 2] try to minimize the impact of this second hit. Unfortunately, to date, the expected benefit (decreased additional tissue damage) of these minimally invasive techniques [3] has not been objectively measurable. The goal of this study is to measure and compare the systemic inflammatory response of patients with pertrochanteric fracture who were operated with two different techniques (DHS conv or DHS MIO). Cytokine levels were used as markers of inflammatory response with the hypothesis that fixation by DHS MIO technique should produce lower levels of circulating cytokines compared with conventional technique, based on lower local tissue trauma.

## Materials and methods

Data from 68 patients were prospectively collected and analyzed after approval of the study plan by the review board and with written consent by all patients enrolled in the study, according to the Declaration of Helsinki.

All patients operated with gliding hip screw (DHS) were included in the study. Thirty-six were operated with conventional technique by two surgeons (DHS conv) and thirty-two with minimally invasive technique by another surgeon (DHS MIO), according to routine surgeons’ turnover in the operating room, thus identifying almost randomly two cohorts of patients.

Patients with cardiovascular diseases needing treatment before surgery according to the anesthesiologist, and patients using steroids, were excluded from the study. (One patient affected by rheumatoid arthritis was excluded from the study because of long-term use of steroids, because of the direct interference of this kind of drug with interleukins.)

Age, comorbidities, medication, AO type of fracture based on conventional X-rays (anteroposterior and lateral), time since accident, length of incision, possible intraoperative complications, and transfusions were recorded.

The minimally invasive approach basically consists in sliding the plate under the vastus lateralis muscle through a small incision, without dissection of its origin. The plate is placed on top of the cephalic screw without use of the guide shaft, and the plate screws are placed through short stab incisions.

Transfusion was performed if the subject had Hb values <9 g/dl and/or hematocrit <27 %.

In both groups, the levels of interleukins IL-6, IL-10, IL-8, and TNF-α were measured 1 h before and 1 h after surgery.

We divided the fractures into three groups: A1, A2, and A3, according to the AO classification.

Different types of implant constructs were used:

- Short plates (two or three holes), type 1
- Longer plates plus antirotation screw, cerclage or +additional trochanteric stabilization plate (ATSP), types 2 and 3.

Length of hospitalization is not documented as it depends on the social situation and availability of rehabilitation beds.

### Statistical analysis

The Student *t* test was applied for continuous variables and chi-square test for discrete quantitative variables, testing significance between variables of the two groups.

The multivariate logistic regression with backward method was performed for IL-6, IL-10, TNF-α, and age. IL-8 was not considered because it was measured in only 36 patients. Using logistic regression we investigate the kind of relationship between variables.

The chi-square test (likelihood ratio) was used to analyze possible significant differences in IL-6 levels depending on type of fixation and severity of fracture. This analysis was limited to IL-6 because it was the only substance showing significantly different levels.

The *F* test was performed to verify the hypothesis that IL-8 levels return to below threshold (32 pg/ml) after surgery without differences between the groups.

## Results

Patient data collected prior to surgery showed no significant differences between the two groups in general characteristics such as sex, number of comorbidities, fracture type, preoperative levels of interleukins (except IL-10), time between hospitalization and surgery, use of oral anticoagulants, and antiplatelet drugs (Table 1).

**Table 1:** Preoperative characteristics of patients

	Conventional (36 subjects)	MIO (32 subjects)	Average difference (95 % CI)	*p* Value
Age (years)	84.7 ± 7.20	82.78 ± 7.71	1.91 (−1.69 to 5.52)	0.2938
Type of fracture	1.89 ± 0.67	2.00 ± 0.57	−0.11 (−0.41 to 0.19)	0.4650
Time from hospitalization to surgery (h)	57.58 ± 28.04	56.97 ± 52.76	0.61 (−19.53 to 20.76)	0.9516
Anticoagulant/antiplatelet^a^	0.47 ± 0.51	0.37 ± 0.49	0.09 (−0.14 to 0.34)	0.4260
Number of comorbidities	2.33 ± 1.35	2.56 ± 1.24	−0.23 (−0.86 to 0.40)	0.4713

There are no significant differences between the two groups (Student *t* test)

^a^One patient used oral anticoagulants (coumarin) and 12 used oral antiplatelet drugs in the MIO group. Two patients used oral anticoagulants and 17 used oral antiplatelet drugs in the DHS conv group

One intraoperative heavy bleeding from the bone occurred in one patient who made use of antiplatelet agents in the DHS MIO group.

Length of incision and need for transfusions (according to the indications defined above) were significantly different between the two groups (Table 2).

**Table 2:** Perioperative data

	Conventional (36 subjects)	MIO (32 subjects)	Average difference (95 % CI)	*p* Value
Length of incision (cm)	11.65 ± 2.64	7.05 ± 1.77	4.61 (3.50 to 5.71)	**<0.0001**
Duration of surgery (min)	56.67 ± 22.42	54.84 ± 15.94	1.82 (−7.71 to 11.35)	0.7038
Type of fixation	1.89 ± 0.67	2.00 ± 0.57	−0.11 (−0.41 to 0.19)	0.4650
Units transfused	2.22 ± 0.99	1.09 ± 1.20	1.13 (0.59 to 1.66)	**<0.0001**

Bold values indicate significant differences found for length of incision and units transfused (Student *t* test)

Neither duration of surgery nor type of fixation showed significant differences between the two groups, as described above (Table 3).

**Table 3:** Chi-square test for discrete quantitative variables

	Conventional (36 subjects)	MIO (32 subjects)	*p* Value^a^
Sex			
Men	4 (30.8 %)	9 (69.2 %)	0.0771
Females	32 (58.2 %)	23 (41.8 %)	
Type of fracture			0.4442
A1	10 (66.7 %)	5 (33.3 %)	0.5383^b^
A2	20 (47.6 %)	22 (52.4 %)	0.2087^c^
A3	6 (54.5 %)	5 (45.5 %)	0.6853^d^
Type of fixation			0.3218
Type 1 (2–3 holes)	7 (38.9 %)	11 (61.1 %)	0.2866^b^
Type 2 (4 holes + ATSP/antirot. screw/cercl.)	12 (63.2 %)	7 (36.8 %)	0.1454^c^
Type 3 (5 or more holes/+ATSP/antirot. screw/cercl.)	17 (54.8 %)	14 (45.2 %)	0.5668^d^
Anticoagulant/antiplatelet	17 (58.6 %)	12 (41.4 %)	0.4184

Some variables such as sex, type of diagnosis, type of fixation, and taking anticoagulant/antiplatelet drugs were grouped into discrete quantitative variables, for which we performed the chi-square test to assess significant differences between the two groups. There were no significant differences between groups for these variables

^a^Chi-square with two degrees of freedom in a 3 × 2 contingency table. Chi-square breakdown to compare the classes of covariates:

^b^First class with third class

^c^First class with second class

^d^Second class with third class

The preoperative levels of IL-6 recorded showed no significant differences between the two groups, whereas postoperative values were on average higher in the DHS conv group (78.41 ± 67.04 pg/ml) compared with the DHS MIO group (39.02 ± 37.36 pg/ml), with average difference of 39.39 pg/ml (95 % CI 12.65–66.13 pg/ml; *p* = 0.0045) (Table 4).

**Table 4:** Levels of interleukins pre- and postoperatively

	Conventional (36 subjects)	MIO (32 subjects)	Average difference (95 % CI)	*p* Value
IL-6 preoperatively	39.57 ± 47.77	25.21 ± 29.73	14.37 (−5.19 to 33.92)	0.1472
IL-6 postoperatively	78.41 ± 67.04	39.02 ± 37.36	39.39 (12.65 to 66.13)	0.0045
IL-8 preoperatively	244.69 ± 476.18	248.57 ± 518.68	−3.88 (−337.20 to 329.48)	0.9813
IL-8 postoperatively	32.88 ± 7.40	73.91 ± 105.47	−41.04 (−87.70 to 5.63)	0.0829
IL-10 preoperatively	4.51 ± 3.19	2.89 ± 1.55	1.62 (0.38−2.86)	0.0110
IL-10 postoperatively	11.27 ± 19.74	8.90 ± 24.89	2.37 (−8.45 to 13.19)	0.6634
TNF-α preoperatively	30.56 ± 30.19	26.38 ± 19.19	4.18 (−8.39 to 16.76)	0.5092
TNF-α postoperatively	39.52 ± 55.13	23.44 ± 17.31	16.08 (−4.54 to 36.70)	0.1241

The table shows the levels of interleukins IL-6, IL-8, IL-10, and TNF-α before and after operation and the comparison between the two groups; values are expressed in pg/ml. IL-6 level shows no significant difference between the two groups in the preoperative phase, whereas it becomes significant in the postoperative period (Student *t* test)

**Fig. 1:**
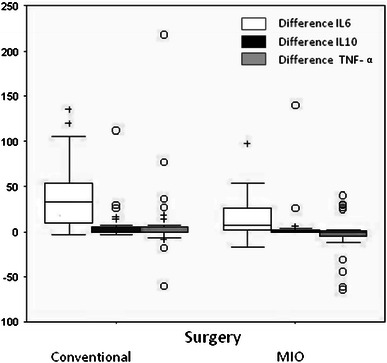
Pre and post-operatively distribution (difference) of IL6, IL10 and TNF-α, for type of surgery

By multivariate logistic regression it was possible to compare the mean differences in levels of interleukins (Fig. 1) before and after surgery, in order to determine the type of relationship and related significance. In all models, IL-6 showed a significant *p* value <0.05. IL-10 and TNF-α did not give significant values.

The chi-square test (likelihood ratio) seemed to show no significant difference between different fracture types for levels of IL-6. For the type of implant, IL-6 levels showed levels of significance just below the threshold (considering 0.05 as the limit). It seems that the levels increased with increase of fixation material (cerclage, ATSP, longer plate) used (Table 5).

**Table 5:** Level of significance for postoperative IL-6 by type of fixation (chi-square *p* value likelihood ratio)

	*p* Value
Type of fixation	0.2563^a^
Type 1 (2–3 holes)	0.0741^b^
Type 2 (4 holes + ATSP/antirot. screw/cercl.)	0.0475^c^
Type 3 (5 or more holes/+ATSP/antirot. screw/cercl.)	0.1912^d^

^a^Total

^b^Type 1 versus type 3

^c^Type 1 versus type 2

^d^Type 1 versus type 2 + type 3

It is worth mentioning that the IL-8 values, consecutively tested in 36 patients (16 MIO and 20 conventional), fell under the threshold of 32 pg/ml postoperatively in 32 cases, remaining above the threshold in 2 cases for each group (*F* test, *p* < 0.0001).

Preoperative IL-10 levels (tested for all subjects) showed significant differences between the two groups: 11 of 38 patients in the DHS conv group and 6 of 32 patients in the DHS MIO group had preoperative IL-10 values just above the threshold of 3.1 pg/ml, but postoperative values did not show any differences between the groups. TNF-α levels (tested for all subjects) also did not show differences between the two groups.

## Discussion

The physiological response to local tissue damage including to skin, subcutaneous tissue, muscle, and bone induces a local inflammatory and systemic reaction designed to maintain the immune balance of the organism and stimulate repair processes [4, 5].

The first local response aims to repair the trauma suffered by various cells (macrophages, monocytes, endothelial cells, fibroblasts, and synovial cells) capable of activating secretion of inflammatory mediators such as cytokines [6, 7].

Recent studies have shown that the concentration of some cytokines is increased after pertrochanteric fracture and that further increase is observed after surgery [8]. American authors [9] have recently compared levels of interleukins, i.e., creatine kinase (CK) and C-reactive protein (CRP), for two different types of surgical approach to total hip replacement. They found that, despite a significant lower increase of CK level with a “minimally invasive” anterior approach compared with posterior, levels of interleukins did not show significant differences between the two groups. Similar results were found many years ago for lumbar fusions by Korean authors [10]. The limited number of patients might explain why these interesting results, published in a prestigious journal, have not received the attention they deserve.

The choice to run the sampling method described was made both to be certain that there could be no other reasons than the intervention affecting changes in the serum markers examined, given their high sensitivity to both internal and external noxa, and to ensure that sufficient time had elapsed after surgery to detect a notable increase of these markers in circulating plasma [8].

The results of our work seem to indicate that invasiveness can be measured according to the systemic inflammatory response resulting after local tissue damage. Especially encouraging are the results of the IL-6 values: no significant differences between the two groups were found in preoperative values, but after a minimally invasive DHS technique a significantly lesser increase was observed compared with a traditional technique (Fig. 1). However, IL-8 seems not to be useful for our purpose of quantifying surgical tissue damage, the same being true for IL-10 and TNF-α.

The hypothesis that different fracture types might stimulate the systemic inflammatory response in different ways through cytokines has not been confirmed at the considered significance level of 0.05. However, levels of IL-6 would be considered significantly different at a level of 0.1 between subjects diagnosed with A1 compared with A3.

While evaluating this data it must be considered that 62 % of fractures are of type A2, and so the above comparison is limited to a small number of subjects (two groups).

The type of implant construct (plate length, use of cerclage and ATSP) seems to influence the IL-6 level (more material increasing the IL-6 level), but additional research is needed to make definitive statements in this regard.

Given the complex role played by cytokines, results from the literature and the present study seem to permit the assumption that IL-6 levels enable measurement of not only local tissue trauma/invasiveness but also the subsequent systemic response.

Proinflammatory cytokine secretion is counterbalanced by anti-inflammatory cytokines [11], thus creating a regulatory system of biological homeostasis that plays the role of an independent predictor of postoperative outcomes in elderly patients suffering fracture (mortality and complications) [12]. Patients with severe cardiovascular morbidity needing preoperative treatment were primarily excluded from this study. It has to be considered that a “high” level of cytokine could also indicate a “proinflammatory state” in elderly patients with no fracture but high cardiovascular risk and morbidity [13].

Locally, cytokines play a key role in the process of repair of many tissues, but their precise role during the healing of fracture remains unknown.

In experimental studies, it has been shown that cytokines (IL-6, IL-1, and TNF-α) exert effects on skeletal homeostasis [14, 15], and in particular IL-1β stimulates proliferation of osteoblasts and production of mineralized bone matrix, and inhibits proliferation and differentiation of chondrocytes. It has also been reported that levels of IL-1β are higher in stabilized than nonstabilized fracture of the tibia, in mice [16].

In 32 patients treated with DHS for pertrochanteric fracture, we found that IL-8 was drastically and significantly reduced after stabilization, but the precise meaning of this phenomenon is not yet certain. The reason for measuring IL-8 was due to recent findings in literature [8]. The authors decided to add this test (without changing anything in the methodology) after the beginning of the work, so the level of IL-8 was collected in only 36 subjects.

Intramedullary nailing is often considered less invasive than plating, because the fracture zone is usually not opened. Further studies, including nail fixation of trochanteric fracture, are ongoing and are needed to confirm the results of this study.

In conclusion, in the treatment of pertrochanteric fracture, secretion of inflammatory markers, especially IL-6, is less marked when minimally invasive techniques are used compared with traditional surgery.

The average patient with pertrochanteric fracture presents numerous comorbidities which may contribute to the finding of elevated preoperative IL levels.

It has to be considered that a further—even if transitory—increase of interleukin levels, due to the surgery, can greatly increase exposure to the risk of complications and morbidity.

By measuring the invasiveness of the intervention (second hit/additional tissue trauma) based on levels of interleukins, it could become possible not only to detect local damage but also to obtain an independent predictor of risk/outcome in elderly patients with pertrochanteric fracture.
